# Cytotoxic and Apoptotic Effects of *Luffa Cylindrica* Leaves Extract against Acute Lymphoblastic Leukemic Stem Cells

**DOI:** 10.31557/APJCP.2020.21.12.3661

**Published:** 2020-12

**Authors:** Shimaa Yehia, Ibrahim M. Abdel-Salam, Basma M. Elgamal, Basma El-Agamy, Germine M. Hamdy, Hala M. Aldesouki

**Affiliations:** 1 *Biochemistry Department, Faculty of Science, Ain Shams University, Cairo, Egypt. *; 2 *Cancer Biology Department, National Cancer Institute, Cairo University, Cairo, Egypt. *; 3 *Clinical Pathology Department, National Cancer Institute, Cairo University, Cairo, Egypt. *

**Keywords:** Luffa cylindrical, Leukemic stem cells, acute lymphoblastic leukemia, CD34/CD38

## Abstract

**Background:**

Acute lymphoblastic leukemia (ALL) is an aggressive malignancy defined by accumulation of lymphoblasts in the bone marrow. Leukemic stem cells (*LSCs*) are the major cause of the recurrence and metastasis of ALL. This study aimed to develop an effective anti-cancer agent targeting these *LSCs*. *Luffa Cylindrica* (*L.C.*) leaves extract was selected to evaluate its effect on ALL via eradicating the *LSCs* as it contains many active anti-cancer flavonoids.

**Methods:**

Thirty-two bone marrow samples of ALL patients were used in this study. *LSCs* population was identified in the selected samples. Cell viability was measured by MTT assay and flow cytometry. Cell cycle, apoptosis, proliferation marker; ki-67 and colony forming assay were further analyzed.

**Results:**

This study revealed the expression of CD34+/CD38+ cells in addition to CD34+/CD38- population and the extract was effective against the two *LSCs* populations. MTT assay showed that treated leukemic cells exhibited significant reduction in the viable cells in a dose dependent manner with IC50 of 3 µg/µl which was then confirmed by flow cytometry. Cell cycle analysis results showed significant reduction in the percentage of cells treated with *L.C.* extract in both the S and G0/G1 phases, with concomitant increase in the G2/M phase. Also, *L.C.* extract could effectively induce apoptosis, inhibit proliferation and suppress colonogenecity of leukemic cells.

**Conclusion:**

This study validated the medicinal potential of *L.C.* leaves extract as a promising anti-leukemic agent targeting both *LSCs* and blasts in ALL patients, which may be explained by the synergy found between its potent flavonoids especially apigenin, luteolin and kaempferol.

## Introduction

Leukemia, the blood or bone marrow cancer was resulted from the overproduction of numerous numbers of abnormal white blood cells (namely called blasts). These immature white blood cells become unable to fight infection and consequently impair the ability of bone marrow to produce the red blood cells and the platelets (Melissa and Jerry, 2017). Acute lymphoblastic leukemia (ALL), the second most common acute leukemia in adults is a malignant transformation and abnormal proliferation of lymphoblasts in the bone marrow (Machado et al., 2017). 

Cancer stem cells (CSCs) are known as minor sub-population of cancer cells with stem cell-like properties, having the ability to generate all types of cancer cells (Francesco et al., 2018). Leukemic stem cells (*LSCs*) play a pivotal role in the incidence, drug resistance, and relapse of the disease (Jiang et al., 2017). *LSCs* are regulated by critical surface antigens like CD34 and CD38 proteins. These proteins are expressed in most cases of leukemia, and hence are used as specific cell markers in both diagnosis and prognosis of the disease (Jiang et al., 2016). So, targeting these cells will provoke a great revolution in the cancer therapy. 

The most medicinal herbs remain safe and alternative effective treatment for many types of cancer, including leukemia (Kabeel et al., 2018). This is due to their huge bioactive constituents (Bashmani et al., 2018). Thus, plant-derived compounds that trigger apoptosis account as promising agents in cancer treatment, especially, CSCs (Claire and Amareshwar, 2018).

Luffa Cylinderica , commonly called sponge gourd, loofa, vegetable sponge or bath sponge, belongs to Cucurbitaceae family is a tropical or sub-tropical and warm climate fast growing plant in high temperature countries such as Asia and Africa. It is used in many Chinese medicine formulations and found all over the world. *L.C.* plant was the herb of choice in this study due to the efficacy of its different parts in the treatment of various types of diseases, besides its antitumor activities (Verma and Rajbala, 2018). The main constituents of the aqueous-ethanol extract of *L.C.* leaves include apigenin 7 glucuronide, eriodictyol -7 glucoside, kaemferide, luteolin - O - diglucoside, neodiosmin, diosmin, kaempferol 3 - [2’’’,3’’’,4’’’ -triacetyl - α - L -arabinopyranosyl -(1 -6) -glucoside] and Lucyoside (Abdel-salam et al., 2018). Its medicinal properties of *L.C.* leaves extract may be due to the presence of apigenin and luteolin which are the crucial flavonoids of the leaves (Onyegbule et al., 2018). Therefore, this present study was intended to determine the cytotoxic and apoptotic effects of the aqueous-ethanol extract of *L.C.* leaves on the *LSCs* as well as ALL blasts.

## Materials and Methods

*Subjects*

The present study was performed on thirty-two bone marrow samples collected from newly diagnosed Egyptian patients (22 males and 10 females) with ALL. Samples were provided from the clinical pathology department of the National Cancer Institute (NCI), Cairo University, Egypt. Past history of malignancy, chemotherapy or radiotherapy as well as chronic diseases or viral infections are among the criteria excluded from our selected subjects. Clinical features of the ALL patients are listed in (Table 1).

The current study was approved by the Institutional Review Board (IRB) of National Cancer Institute, Cairo University, Egypt and IRB No.: IRB00004025, Approval No.: 201617052.4, Issue Date: 04 July 2018. Informed consent follows the criteria of the World Medical Association Declaration of Helsinki. 

*Primary samples preparation*

Mononuclear cells were isolated from the bone marrow samples of newly diagnosed patients with ALL (n=32) by density gradient centrifugation using lymphoprep. (Ficoll/Hypaque) with specific gravity: d=1.077 CE (Gibco, Cairo, Egypt) and cultured in Roswell Park Memorial Institute medium (RPMI1640) supplemented with 10% fetal bovine serum, 1% L-glutamine (Gibco, Cairo, Egypt), 100U/ml Penicillin and 100 µg/ml streptomycin in a 5% CO_2_ –humidified incubator. 

*Luffa cylindrica leaves extract preparation*

*L.C.* leaves were obtained from Orman garden, Giza, Cairo, Egypt; they were authenticated by prof. Dr Abdel Salam El Noyehy, Professor of Taxonomy, Botany Department, Faculty of Science, Ain Shams University, Cairo-Egypt. Voucher specimens were deposited at the herbarium of pharmacognosy department, Faculty of Pharmacy, Ain Shams University, Cairo-Egypt under code (PHG-P-LC-251). *L.C.* leaves were collected and washed and immersed in 1% acetic acid for 15 minutes and rewashed. The leaves of *L.C.* were dried, ground, and then extracted successively by aqueous-ethanol solvent (1:1) for 72 h. with occasional shaking. The extract was filtered once, and the filtrate was evaporated at RT to obtain the concentrated extract. The extract was collected in amber vials and kept at 4^o^C until being used.

*Cell viability assays*

*MTT Cell viability assay*

The cytotoxic effect of *L.C.* leaves extract was evaluated against leukemic cells using 3-(4,5-dimethylthiazol-2-yl)-2, 5-diphenyltetra-zolium bromide (MTT) assay (Sigma –Aldrich) colorimetric assay. Briefly, cells were plated at a density of 5.0 ×10³ cells/well in 96-well culture plates and then treated for 24 h. with four different doses of the extract; 0.5, 1, 2 and 3 µg/µl. After treatment, 20 µL of the MTT solution (5.0 mg/ml in PBS) were added to each well and incubated for 2 h. MTT formazan was dissolved in 150 µL dimethyl sulfoxide (DMSO) and then, the absorbance was measured at 595 nm with an ELISA reader (Tecan Group Ltd, Männedorf, Switzerland). The relative viability of the treated leukemic cells was expressed as percentage of the untreated ones. 

*Flow cytometric viability assay using 7-Amino-Actinomycin D dye*

In order to confirm the cytotoxic and anti-proliferative effects of *L.C.* leaves extract on the leukemic cells, flow cytometric viability assay was conducted using the 7-Amino-Actinomycin D (7-AAD) dye. Briefly, 20 µL of the 7-AAD viability dye solution (Beckman Coulter Inc., Cairo, Egypt) were added to the 100 µL of approximately 5×10^3^ cells, vortexed gently, incubated for 15 to 20 min at RT and protected from light. After washing, the labeled cells were analyzed within 1hour by flow cytometer. 

*Cancer stem cells identification by flow cytometric analysis*

CSCs populations were identified in the selected bone marrow samples of ALL via the flow cytometric analysis. At least 200,000 cells were tested for fluorescent labeled monoclonal antibodies and respective isotope controls. Briefly, 10 µl of specific conjugated antibody (anti-human CD38 isothiocyanate (CD38-FITC; Beckman Coulter Inc., Cairo, Egypt) and anti-human CD34 phycoerythrin (CD34-PE; Beckman Coulter Inc., Cairo, Egypt) were added to 100 µl of cells, vortexed gently, incubated for 15 to 20 min at dark place. After washing the cells with 1 ml of PBS, the labeled cells were analyzed by flow cytometer. All data were analyzed using Diva 6.1.1 software. 

*Cell cycle analysis*

Cell cycle analysis was carried out to detect the probable changes in the cell cycle phases before and after treatment with *L.C.* leaves extract. Briefly, 106 cells were suspended in 0.5 ml PBS and fixed in 70% ethanol on ice. The ethanol-suspended cells were centrifuged, the cell pellet was centrifuged again and resuspended in 1 ml of propidium iodide (PI) staining solution and kept in the dark at RT for 30 min. Then, the cell fluorescence was measured (Beckman Coulter Inc., Cairo, Egypt) for cell cycle analysis. The percentage of cells in GO/G1, S, and G2/M phases of the cell cycle was calculated using Cell Lab Quanta SC software.

*Cell apoptosis analysis*

The apoptotic activity of *L.C.* leaves extract on leukemic cells was evaluated by flow cytometry using Annexin V-FITC and propidium iodide (Beckman Coulter Inc., Cairo, Egypt). 10^6^ Cells were treated with *L.C.* extract (3 µg/µl) for 24 h. and washed with PBS prior to centrifugation. The cell pellets were resuspended in ice-cold 1X binding buffer. The cells were stained in propidium iodide (PI) and Annexin V-FITC solution. The cell suspensions were kept on ice and incubated for 15 min in the dark. Cells were then analyzed by flow cytometry. The percentage of the apoptotic cells was determined by the flow cytometric analyzer. 

*Proliferation marker Ki67 analysis*

In order to assess the anti-proliferative effect of *L.C.* leaves extract, Ki67 proliferation marker was investigated using ELISA Kit for Ki-67 protein (Cloud- Clone Crop., USA, no. ABIN415150) to assess the proliferation of cultured cells in vitro. 

*Colony forming assay*

The impact of *L.C.* leaves extract on the self-renewal and differentiation capabilities of CSCs in vitro was assessed by the colony forming assay. The cells (3.0 ×10^4^) were suspended in 1.6 ml RPMI agarose medium (10% FBS and 0.33% agarose) containing DMSO, plated in each well of a 6-well plate and poured over a lower layer of solidified RPMI agarose medium (10% FBS and 0.5% agarose). Cultures were maintained for 2.0 weeks without fresh medium feeding at 37◦C in a humidified atmosphere of 95% air and 5.0% CO_2_, after which cell colonies > 0.1 mm were enumerated and photographed. 

*Effect of L.C. on cell morphology*

The bone marrow samples selected in this study were stained with Leishman dye. The morphological apoptotic changes were evaluated before and after treatment using oil immersed lens of light microscope.

*Statistical analysis*

Statistics was performed by Paired-Sample T-Test for comparison between the treated and untreated cells using SPSS (Statistical Package for Social Science), version 23 and Microsoft Excel program. All Numerical data was presented as mean ± S.E form at least three independent experiments. P-Values < 0.05 were considered statistically significant.

## Results

*Cell viability assays*

*Cytotoxic effect of L.C. leaves extract on the leukemic cells*

**Figure 1:**
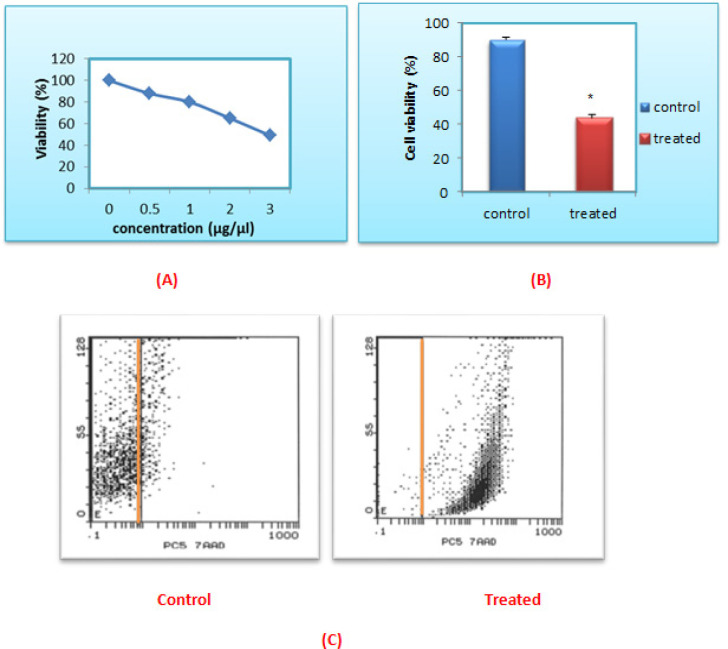
The Cytotoxic Effect of Aqueous-Ethanol Extract of *L.C.* Leaves on Cell Viability of ALL Cells. (A) IC_50_ of *L.C.* leaves extract on ALL cells was measured by MTT assay. (B) The effect of L.C. leaves on cell viability of ALL cells by using by 7-AAD assay. The data was normalized to the untreated group: *P<0.001. Data was expressed as mean ± SEM from 32 independent experiments. (C) Fluorescence intensity vs. side scatter biparametric histogram showing the viable cells without 7-AAD viability dye on the left side and the non-viable cells with 7- AAD viability dye on the right side

The present results showed that the 100% cell proliferation of the leukemic cells was significantly reduced in a dose-dependent manner to 88.27%, 80.79%, 65.53% and 50% upon treatment with 0.5, 1, 2 and 3 µg/µl of *L.C.* leaves extract respectively. Accordingly, the obtained IC_50_ value was 3 µg/µl as shown in (Figure 1A).

*Flow cytometric viability assay using 7-AAD dye*

The current flow cytometric results showed that the viability of the 24 h. post treated leukemic cells with the *L.C.* leaves extract was significantly reduced from 89.99±1.96% to 43.92±5.59% (P<0.001), compared to the untreated control ones as shown in (Figure 1B and C). 

*Cancer stem cells identified by Flow cytometric analysis*

**Figure 2:**
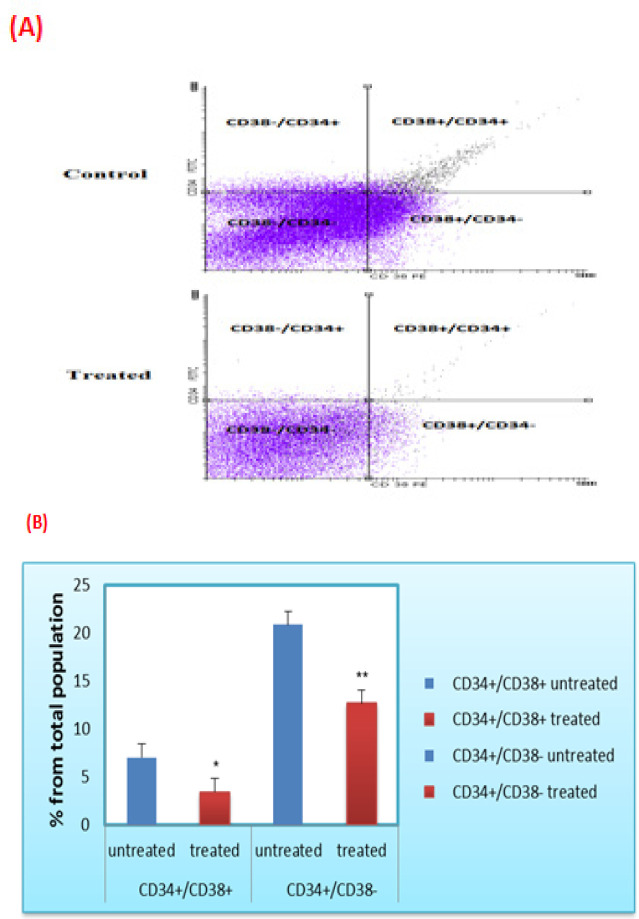
Cytotoxic Effect of *L.C.* Leaves Extract on the LSCs. (A) Flow cytmetry dot plot chart represents CD38/CD34 ratio before and after treatment with the extract. The first and second quadrants represent acute lymphoblastic leukemic stem cells. (B) The ratio of CD34+/CD38+ and CD34+/CD38-populations was evaluated by flow cytometry before and after treatment with the extract. Data was expressed as mean ± SEM (* P<0.05) and (** P<0.01) compared to the untreated cells

The current findings identified the presence of two CSCs populations (CD34+/CD38+ and CD34+/CD38-) in the studied ALL bone marrow samples. Additionally, the flow cytometric results revealed a significant decline in the CD34+/CD38+ cells from 7.05±2.40% to 3.45±0.91% (P<0.05) in the treated samples. Consistently, CD34+/CD38- cells were significantly reduced from 20.89±7.88 % to 12.7±4.77% (P<0.01) upon treatment with *L.C.* herbal extract, compared to the untreated ones. These results confirmed the efficacy of *L.C.* extract against both leukemic stem cell populations. Results also indicated that 60% of the CD34+/CD38+ and 20% of CD34+/CD38- cell populations respond well to the herbal extract (Figure 2).

*Luffa cylindrica leaves extract induces cell cycle arrest*

**Figure 3:**
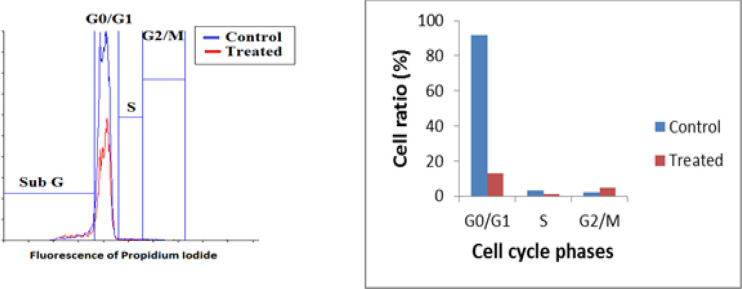
Cell Cycle Analysis of ALL Cells Treated with *L.C*. Leaves Extract Compared to the Untreated Control Cells

As shown in (Figure 3), a significant decrease in the G0/G1 phase from 92% to 13.2% (p< 0.05) was detected in the 24 h. post treated leukemic cells with *L.C.* leaves extract as compared to the untreated cells. Consistently, the percentage of the cells in the S phase showed a significant decline from 3.03% to 1.12% (p<0.05) in the treated leukemic cells, compared to the untreated ones. Controversy, the percentage of the *L.C.* post treated leukemic cells in the G2/M phase was significantly increased from 2.21% to 4.94% (P<0.05) as compared to the untreated leukemic cells. 

*The apoptotic impact of L.C. leaves extract on leukemic cells*

**Figure 4:**
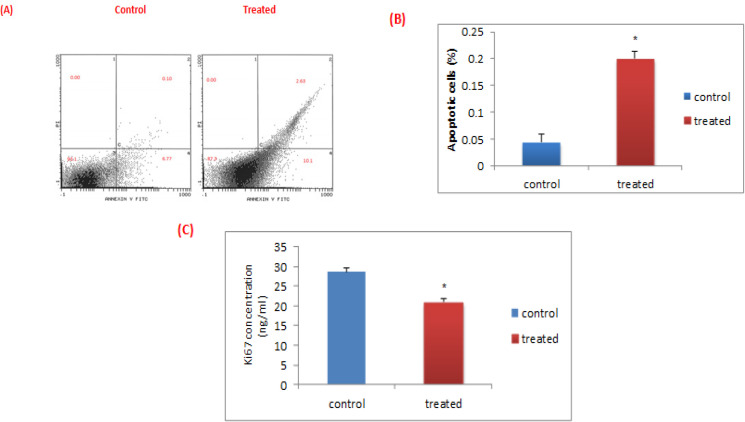
Apoptosis and Proliferation in ALL Bone Marrow Samples before and after Treatment with *L.C *Leaves Extract. (A) Flow cytometric dot plot chart by Annexin V and Propidium Iodide in the untreated and the treated leukemic cells. (B) The percentage of apoptotic cells was evaluated by flow cytometric analysis before and after treatment with the extract. Data was expressed as mean ± SEM (* P<0.05) compared to the untreated cells. (C) Effect of* L.C.* leaves extract on ki-67 protein concentrations in leukemic cells

Flow cytometric results showed that the treatment of leukemic cells with aqueous-ethanol extract of *L.C.* leaves for 24 h. significantly induces apoptosis in ALL cells compared to the untreated ones (0.20 ± 0.05% versus 0.04 ± 0.01 %, P<0.05). Notably, in the treated leukemic cells, the percentage of viable cells in the third quadrant significantly decreased from 93.1% to 87.3% compared to the untreated ones. Moreover, the late apoptotic cells in the second quadrant are significantly increased from 0.10 % to 2.63%. Similarly, the early apoptotic cells in the fourth quadrant are significantly increased from 6.77% to 10.1%. While, there is no change in the percentage of the necrotic cells in the first quadrant before and after treatment (Figure 4 A and B).

*The anti-proliferative effect of the L.C. leaves extract*

The present results showed that the concentration of the proliferation marker, Ki-67 was significantly decreased from 28.70 ± 6.27 to 20.99 ± 5.15 (P<0.01) in the *L.C.* treated cells, compared to the untreated leukemic ones, as shown in (Figure 4C).

*Effect of L.C. leaves extract on Clonogenicity*

**Figure 5:**
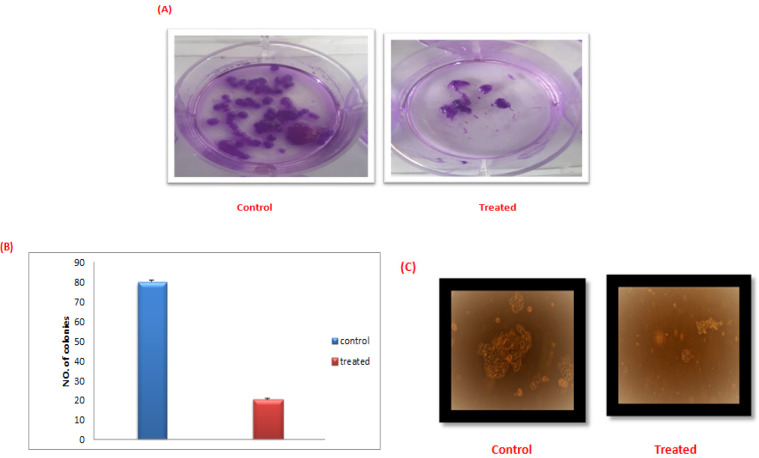
Cytotoxicity of *L.C*. Leaves Extract on the LSCs. (A), (B) Colonies’ number before and after treatment with the extract. (C) Decline of colonies size after treatment with the extract under inverted microscope

The preset results showed a detectable reduction in the colony forming ability of the leukemic stem cells upon treatment with *L.C.* leaves extract. Notably, the colonies’ numbers as well as size were reduced in the treated cells compared to the untreated ones as shown in (Figure 5). 

*L.C. leaves extract induces morphological apoptotic changes*

**Figure 6:**
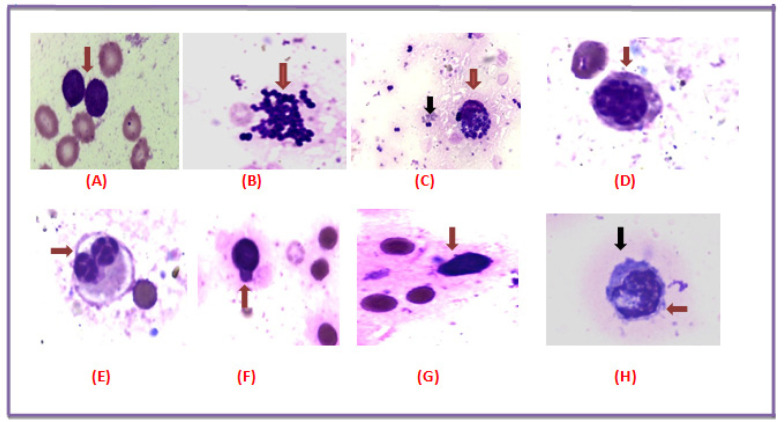
*L.C*. Leaves Induced Morphological Apoptotic Changes in ALL. (A) Bone marrow smear aspirate showing a leukemic blast cell without treatment. (B) Released apoptotic bodies from a broken cell. (C) Red arrow shows formation of apoptotic bodies in a blast cell and Black arrow shows scattered apoptotic bodies. (D) Formation of apoptotic bodies in a large leukemic blast cell. (E) Red arrows show a large binucleated leukemic blast cell with characteristic apoptotic bodies. (F) Blast shows distinct cytoplasmic bleds or psedopods formation. (G) Red arrow shows condensation of the nuclear chromatin along the nuclear envelope of a blast cell. (H) Red arrow shows Formation of apoptotic bodies in a blast cell and Black arrow shows a leukemic blast with characteristic cytoplasmic blebbing

The impact of *L.C.* leaves extract on the morphology of all studied bone marrow films revealed the apoptotic changes compared to the untreated leukemic blasts. These observed changes including released apoptotic bodies from a broken cell, scattered apoptotic bodies, formation of apoptotic bodies in a blast cell, blebbing of cytoplasmic membrane and condensation of the nuclear chromatin along the nuclear envelope as identified under light microscope as shown in (Figure 6A-H).

**Table 1:** Clinical Features of the ALL Patients

Clinical parameters	Patient number (n=32)	Proportion (%)
Age, median (range), y	28 (1-60)	-
1-20 years, No., (%)	10	31.25%
21-40 years, No., (%)	12	37.5%
41-60 years, No., (%)	10	31.25%
WBC count, median (range), × 10^9^/L	2.03 (1.1-129.8)	-
Hemoglobin, median (range), g/dL	8.3 (6.2-11.2)	-
Platelet count, median (range), × 10^9^/L	41 (4-821)	-
Blasts in BM (%), median (range)	80 (10-97)	-
≤ 50%, No., (%)	10	31.25%
51-80%, No., (%)	6	18.75%
≥ 81%, No., (%)	16	50%
Gender		
Male, No., (%)	22	68.75%
Female, No., (%)	10	31.25%

Abbreviations: ALL, acute lymphoblastic leukemia; BM, bone marrow; WBC, white blood cell.

## Discussion

Acute lymphoblastic leukemia (ALL) is a life-threatening cancer, characterized by accumulation of lymphoid blast cells in hematopoietic tissues, especially the bone marrow. Leukemic stem cells (*LSCs*), being the cancer-initiating cells are the main cause of disease relapse. Hence, they are an evolving target of anti-leukemia treatment. So, the development of anti-*LSCs* agents as new therapeutic remedies was needed to improve the patient’s prognosis and to reduce the leukemic-related mortality (Erebiá and Waiá, 2015). 

Luffa cylindrica (*L.C.*) with its different parts have shown powerful biological activities (Hlel et al., 2017). The cytotoxic activity of the whole plant ethanol extract on the HT-29 and HCT-15 cell lines has been reported (Sharma et al., 2015). In addition, the *L.C.* seeds have proven anti-tumor activity due to its active constituent, Luffin (Liu et al., 2010). Moreover, aqueous-ethanol herbal extract of *L.C.* leaves has shown effective anticancer activity against different breast cell lines (Abdel-Salam et al., 2018). Therefore, the current study aims to evaluate the therapeutic ability of *L.C.* leaves extract by targeting the blasts and the *LSCs* sub-populations identified and expressed in ALL patients. 

HPLC/MS analysis of the aqueous-ethanol extract of *L.C.* leaves revealed the presence of various bioactive constituents. The most abundant two flavonoid compounds are apigenin and luteolin which used as key components of *L.C.* leaves extract as reported by Abdel-Salam et al., 2018. However, the MTT assay results confirmed the safety as well as the cytotoxic effect of *L.C.* leaves extract. These findings may be due to the presence of apigenin, luteolin and kaempferol in the extract which decreased the cell viability in human leukemia cells as previously reported by Jayasooriya et al., (2012); Wang et al., (2018); Moradzadeh et al., (2018), respectively.

The identification of CSCs has potential therapeutic modulations. In ALL, CD34+/CD38- cell population was previously identified (Cobaleda et al., 2000). Recently, CD34+/CD38+ CSCs were also shown to be expressed (Blatt K. et al., 2018). In line with, our results identified the expression of the two different CSCs population; CD34+/CD38+ and CD34+/CD38- in the Egyptian patients with ALL. Noteworthy to note that the *L.C.* extract was effective against both CSCs sub-populations. It has been suggested that CSCs are linked with apoptotic pathway, due to their ability to overexpress anti-apoptotic genes such as Bcl-2. Thus, the cytotoxic and apoptotic-inducing effects of the *L.C.* extract may be attributed to its ability to eradicate the two crucial CSCs populations. Moreover, this apoptotic impact was referred to the selective apoptotic-inducing effect of apigenin on the CD34+/CD38- leukemic cells without harming the healthy hematopoietic ones, which can be achieved via the PI3K/AKT pathway inhibition (Cheong et al., 2010) or P53-related apoptotic pathway (Sung et al., 2016; Madunic et al., 2018). In accordance with, apigenin has been previously known to inhibit the self-renewal capacity of *LSCs* in HeLa cells line (Tang et al., 2014; Liu et al., 2014). As well, the potent apoptotic impact of *L.C.* leaves extract was previously confirmed on three different types of breast cell lines (MCF-7, BT-474 and NDA-MB-231) (Abdel-Salam et al., 2018). 

The apoptotic effect of the *L.C.* extract was as well confirmed by its impact on the morphology. As evidenced, a significant reduction in both the colonies’ number and size of the treated *LSCs* was detected. Recently, Abdel-Salam et al, reported that the hot water extract of the whole *L.C.* plant exhibited significant decrease in the sphere’s diameter of the circulating cancer stem cells in hepatocellular carcinoma (Abdel-Salam et al., 2019). It has been shown that apigenin, significantly inhibited the stemness features of the triple-negative breast cancer (TNBC) cells and reduced the rate of colony formation in the TNBC cell lines. Similarly, Luteolin, was known to suppress the stemness of prostate cancer cells by inhibiting the Wnt signaling via transcriptional upregulation of frizzle class receptor 6 (FZD6) (Li et al., 2018). Kaempferol can induce apoptosis through inhibition of telomerase expression and multidrug resistance protein as well as increasing the Bax/Bcl 2 ratio in leukemic cells (Kashafi et al., 2017; Moradzadeh et al., 2018). 

The significant downregulation in the Ki67 proliferation protein detected in the *L.C.* treated leukemic cells reflects its anti-proliferative effect. The anti-proliferative effect of *L.C.* was accomplished through multiple and complex pathways such as apoptosis, ROS and DNA repair (Salmani et al., 2017). 

The current study confirmed the impact of the *L.C.* extract on the cell cycle arrest, as evidenced by the significant reduction in the G0/G1 and S phases with the concomitant increase in the G2/M phase. Recent studies reported that the bioactive flavonoids can induce the cell cycle arrest via increasing the expression levels of p53 and p21, as well as inhibiting the different cyclins and cyclin-dependent kinases (Saraei et al., 2019). So, the cell cycle arrest detected throughout the study at a specific checkpoint upon treatment with *L.C.* extract may be due to the presence of apigenin, which stimulates P53 accumulation, DNA damage, expression of the pro-apoptotic protein BAX and apoptosis (Meng et al., 2017).

In conclusion, the results of this study demonstrated the potent apoptotic and cytotoxic activities of the aqueous-ethanol extract of the *L.C.* leaves against both *LSCs*, populations which are represented by CD34+/CD38+ and CD34+/CD38- cell populations as well as ALL blasts. In addition, the anti-proliferative effect of the *L.C.* extract was proven by the decrease in the G0/G1 and S phases of the cell cycle as well as the decrease in the expression levels of the proliferation marker, ki67 protein in the treated leukemic cells. These results were confirmed by inhibition of the viability of treated ALL cells and decrease in colony formation ability of *LSCs*. Due to the synergy between the different active flavonoids such as apigenin, luteolin and kaempferol, *L.C.* leaves extract could be a promising herb which can be used as anti-cancer agent targeting *LSCs* and ALL blasts. Further comparative studies needed to be conducted on different extracts of *L.C.* leaves in order to get the most effective anti-cancer agent targeting the CSCs. 
